# Identifying Compound Effect of Drugs on Rheumatoid Arthritis Treatment Based on the Association Rule and a Random Walking-Based Model

**DOI:** 10.1155/2020/4031015

**Published:** 2020-11-06

**Authors:** Yanyan Fang, Jian Liu, Ling Xin, Yue Sun, Lei Wan, Dan Huang, Jianting Wen, Ying Zhang, Bing Wang

**Affiliations:** ^1^Department of Clinical Data Center, The First Affiliated Hospital, Anhui University of Chinese Medicine, Hefei 230031, China; ^2^Department of Rheumatism Immunity, The First Affiliated Hospital, Anhui University of Chinese Medicine, Hefei 230031, China; ^3^Graduate Faculty, Anhui University of Chinese Medicine, Hefei 230038, China; ^4^School of Electrical & Informatics Engineering, Anhui University of Technology, Maanshan 243002, China

## Abstract

Rheumatoid arthritis (RA) is a chronic autoimmune disorder that is diagnosed mainly on the basis of patient signs, symptoms, and laboratory indices. However, the exact causes of RA are unclear. Moreover, there is a lack of any method of dynamically evaluating the efficacy of the medication administered to treat RA. Here, we applied a random walk model to reveal the compatibility among the various constituents of traditional Chinese medicine and evaluate their therapeutic efficacy against RA. Drugs commonly used to treat RA were investigated using cluster analysis. The association rule analysis was applied to identify compatibilities among the constituents. A random walk model was developed to evaluate drug efficacy based on an in-house database comprising the clinical records of 9,408 RA patients. Frequently administered medicines were combined into three correlated sets. The evaluation based on the random walk method showed that the drug combination improved ESR, CRP, C3, C4, and IgA more effectively than any single drug. The present study demonstrated that the TCM constituents complement each other and various combinations of them produce different therapeutic effects on RA treatment.

## 1. Introduction

Rheumatoid arthritis (RA) is a common, refractory systemic autoimmune disease caused by multiple factors [1]. RA morbidity is increasing worldwide. However, it is difficult to treat this condition as its etiology and pathogenesis are unclear [2]. In certain patients, the symptoms cannot be effectively controlled [3]. Several new synthetic and biological disease-modifying antirheumatic drugs (DMARDs) are administered as RA therapy. Nevertheless, these treatments are lengthy and costly and induce adverse reactions [4, 5]. Therefore, it is imperative to find new RA therapies that are efficacious and economical and cause few side effects.

For many years, Chinese herbal medicine has been administered for RA treatment [6, 7]. Combinations of various medications such as oral Chinese herbal decoctions, prescription preparations, and external applications have attracted interest in recent years as they have diverse applications [8–11]. They regulate multiple targets in immunity and inflammation, work synergistically, are unlikely to induce resistance, and may have optimal clinical efficacy [12]. At present, however, there is still no effective clinical evaluation system for Chinese herbal medicine application. Further, relative therapy remains to be investigated in order to determine the rationality and regularity of herb compatibility. Hence, we pursued these research objectives in the present study.

Data mining technology uncovers and compiles potentially valuable knowledge based on a large amount of data. It has been widely used in the field of medicine. This process comprises data preparation and mining followed by expression and analysis of the results. It is mature information processing that involves database applications. Database technology entails data processing and information management. Data are processed in the database and analyzed by studying the basic theory and practice of structure, storage, design, management, and application [13]. Data mining technology includes various methods and extracts different aspects of the information content.

Here, we evaluated the clinical therapeutic effects and the pharmaceutical rule of medicine in the treatment of patients with RA. We used clustering analysis, association rules, a baseline matching algorithm, and a random walk model to analyze clinical RA data. The results showed complementary information among the drugs. Various combinations of them could produce different therapeutic effects in RA treatment.

## 2. Material and Methods

### 2.1. Materials

Hospitalization data were compiled for persons who were RA inpatients between July 2009 and May 2019 in the Department of Rheumatology and Immunology of the First Affiliated Hospital of Anhui University of Chinese Medicine. The dataset consisted of records of the use of Chinese herbal medicine, Xinfeng capsule prescription preparation, Furong ointment, and disease-related laboratory indices including the inflammatory markers CRP and ESR and the immune indicators CCP, RF, IgA, IgM, IgG, C3, and C4. The research scheme was approved by the Ethics Committee of the First Affiliated Hospital of Anhui University of Chinese Medicine. A total of 10,155 patients with RA were searched, of which 9,408 were treated with Chinese herbal medicine. The patients were assigned either to a control group (Chinese herbal medicine alone) or an experimental group (Chinese herbal medicine plus Xinfeng capsule/Furong ointment prescription preparation). There were 3,533 cases in the control group and 5,875 cases in the experimental group.

### 2.2. Methods

#### 2.2.1. Cluster Analysis

The designation for the use of Chinese herbal medicine was 1 while that for nonuse was 0. Chinese herbal medicine compatibility was investigated by systematic clustering in SPSS v. 21.0 (IBM Corp., Armonk, NY, USA). In the clustering analysis algorithm, each herb was regarded as a cluster, and *N* clusters were combined to form a new class based on the similarity between objects. The Euclidean metric was used to calculate similarity between herbs [14]:

(1) $$\begin{array}{c} d\left(x,y\right)=\sqrt{\overset{n}{\underset{k=1}{\sum }}{\left(x_{k}-y_{k}\right)}^{2}}. \end{array}$$

#### 2.2.2. Association Rules

*(1) Apriori Algorithm*. The designation for the use of Chinese herbal medicine was 1 while that for nonuse was 0. The Apriori module in SPSS Clementine v. 11.1 (IBM Corp., Armonk, NY, USA) was used to identify correlations among Chinese herbal medicines. We set the minimum support and confidence to 80% and the degree of improvement to >1. The Apriori algorithm was implemented to establish the relationships among items within a dataset. It is also known as a shopping blue analysis. Each drug was treated as a variable in this dataset. The formulae [14] applied were as follows:

(2) $$\begin{array}{c} \text{support}\left(X⟶Y\right)=\sigma \frac{\left(X\cup Y\right)}{N},\\ \text{confidence}\left(X⟶Y\right)=\sigma \frac{\left(X\cup Y\right)}{\sigma \left(X\right)},\\ \text{expected confidence}\left(X⟶Y\right)=\sigma \frac{Y}{N},\\ \text{lift}\left(X⟶Y\right)=\text{confidence}\frac{\left(X⟶Y\right)}{\sigma \left(Y\right)}, \end{array}$$

where *X*⟶*Y* is an association rule, *X* (left-hand side (LHS)) and *Y* (right-hand side (RHS)) represent the set of herb items, *σ*(*X*) is the frequency of itemset *X*, *X* ∪ *Y* is the union of itemsets *X* and *Y*, *σ*(*X* ∪ *Y*) is the frequency with which itemsets *X* and *Y* appear together, support(*X*⟶*Y*) is the frequency with which *X* and *Y* appear together, and confidence(*X*⟶*Y*) is the probability that itemset *Y*appears in the presence of *X*. Expected confidence(*X*⟶*Y*) is the probability that itemset *Y* appears without any conditional influence. Lift is the ratio of the probability that itemset *Y* appears in the presence of itemset *X* to the frequency of itemset *Y*. Support and confidence are often used to eliminate meaningless combinations. Lift indicates the validity of the association rules.

*(2) FP-Growth Method*. Frequent pattern growth (FP-Growth) adopts a divide-and-conquer technique and recursively projects a transactional database into a set of smaller projected transactional databases and mines frequent itemsets in each projected database by exploring only locally frequent items. This mines the complete set of frequent itemsets and substantially reduces those candidate itemsets that do not exist in the database. FP-Growth stores the transactional database in a highly condensed much smaller data structure called frequent pattern tree (FP-tree). The support of candidate itemsets is counted directly from the FP-tree without scanning the original database multiple times. This improves the processing speed of the algorithm [15]. We set each Chinese herbal medicine as an itemset and explore the frequency between itemsets.

#### 2.2.3. Baseline Matching Algorithm

**Figure 1:**
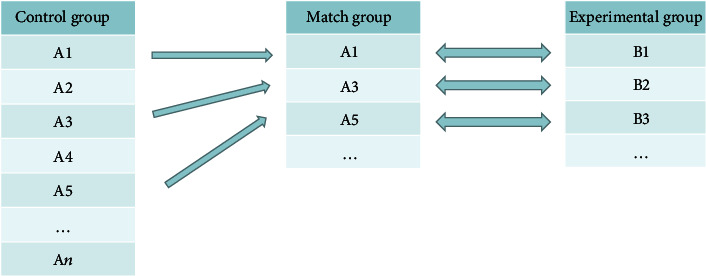
Schematic diagram of baseline matching algorithm.

The baseline matching algorithm solves real-world inconsistencies in patient condition (immune inflammation index). It is based on a 2D Euclidean distance. Starting from its minimum value, the target area is tracked and stripped from small to large, one unit at a time, until the target area is empty. In this way, objects near the target value are obtained. Matching is executed as shown in Figure 1.

#### 2.2.4. Random Walking

The Oracle Developer Suite 10g was used to evaluate the random walking model of the immune inflammation index and observe drug compatibility improvement for the laboratory indices. The concept of random walking was first proposed by Pearson in 1905 to delineate the trajectory of microscopic matter (Wu, 1997). For a conventional one-dimensional random walking model, a walker either moves up (*u*(*i*) = +1) or down (*u*(*i*) = −1) by one-unit length (*u*) for each step *i* of the walk^2^. For an uncorrelated walk, the direction of each step is independent of those of the previous steps. For a correlated random walk, the direction of each step is independent of the history (“memory”) of the walker. A random walk naturally motivates the quantification of this correlation by calculating the “net displacement” (*y*) of the walker after one step which is the sum of the unit steps *u*(*i*) for each step *i* [16]:

(3) $$\begin{array}{c} y\left(l\right)=\overset{l}{\underset{i=1}{\sum }}u\left(i\right). \end{array}$$

An important statistical quantity characterizing any walk^2^ is the root mean square fluctuation *F*(*l*) about the average of the displacement. *F*(*l*) is defined as the difference between the average of the square and the square of the average

(4a) $$\begin{array}{c} F^{2}\left(l\right)=\bar{{\left[\Delta y\left(l\right)-\bar{\Delta y\left(l\right)}\right]}^{2}}=\bar{{\left[\Delta y\left(l\right)\right]}^{2}}-{\left[\bar{\Delta y\left(l\right)}\right]}^{2}, \end{array}$$

of a quantity Δ*y*(*l*) defined by

(4b) $$\begin{array}{c} \Delta y\left(l\right)=y\left(l_{0}+l\right)-y\left(l_{0}\right). \end{array}$$

The output of this operation is equivalent to (1) walking a set of calipers for a fixed distance *l*, (2) sequentially moving the starting point from *l*_0_ = 1 to *l*_0_ = 2 and so on, (3) calculating the quantity Δ*y*(*l*) and its square for each *l*_0_, and (4) averaging all calculated quantities to obtain equation (4a):

(5) $$\begin{array}{c} F^{2}\left(l\right)∼l^{\alpha },\;\text{with }\alpha \neq \frac{1}{2}. \end{array}$$

### 2.3. Statistical Processing

All data were analyzed in SPSS v. 21.0 (IBM Corp., Armonk, NY, USA). A nonparametric test on two related samples was run for the control and experimental groups before and after treatment. Differences between groups before and after treatment were compared with a Mann-Whitney rank sum test. Differences were considered statistically significant at *P* < 0.05.

## 3. Results

### 3.1. Administration of Chinese Herbal Medicine in RA Treatment

A total of 10,155 patients with RA were searched, of which 9,408 were treated with Chinese herbal medicine. Each patient with RA consumed a prescription of Chinese herbal medicine composed of >300 species. The first 20 Chinese herbal medicines were divided into four categories according to their efficacy. These included *Poria*, *Pericarpium Citri Reticulatae*, *Semen Coicis*, *Rhizoma Dioscoreae Oppositae*, and *Fructus Hordei Germinatus* to invigorate the spleen and resolve dampness; *Radix Salviae Miltiorrhizae*, *Flos Carthami*, *Semen Persicae*, *Caulis Spatholobi*, and *Rhizoma Chuanxiong* to promote blood circulation and dredge collaterals; *Radix et Rhizoma Clematidis Chinensis*, *Sigesbeckia orientalis*, *Rhizoma Alismatis*, *Semen Plantaginis*, and *Radix Angelicae Biserratae* to dispel wind and dehumidify; and *Herba Taraxaci Mongolici*, *Herba Hedyotdis*, *Radix Scutellariae Baicalensis*, *Cortex Phellodendri Amurensis*, and *Rhizoma Anemarrhenae* to clear heat and detoxify. According to meridian tropism statistics of Chinese herbal medicine, the maximum number of applications of Chinese herbal medicine pertaining to the spleen meridian is 35,478. According to the taste statistics, sweet taste is administered up to a maximum of 57,052 times while bitter taste is given at the most 50,762 times (Table 1).

### 3.2. Cluster Analysis of Chinese Herbal Medicine in RA Treatment

**Figure 2:**
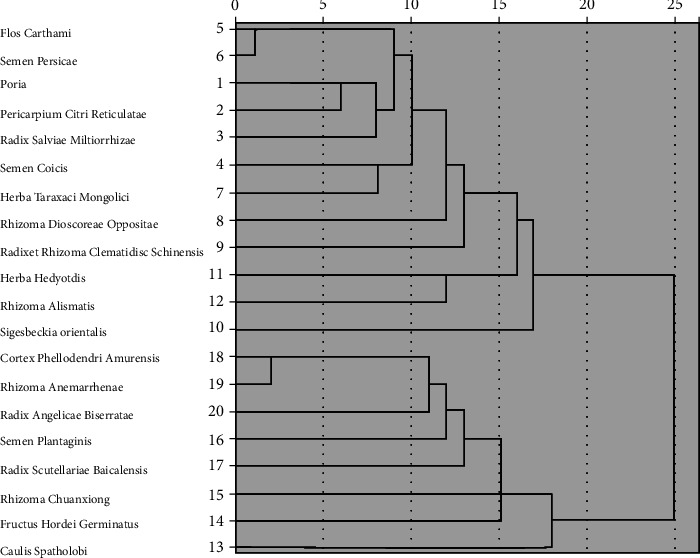
Cluster analysis of Chinese herbal medicine used in RA treatment. Note: when Euclidean distance = 15, Chinese herbal medicines were divided into three sets. Neither *Sigesbeckia orientalis* nor *Caulis Spatholobi* was classified in the other sets.

We ran a cluster analysis of Chinese herbal medicines used to treat RA. At a Euclidean distance of 15, Chinese herbal medicine was further divided into three sets (Figure 2).

Set 1: *Flos Carthami*, *Semen Persicae*, *Poria*, *Pericarpium Citri Reticulatae*, *Radix Salviae Miltiorrhizae*, *Semen Coicis*, *Herba Taraxaci Mongolici*, *Rhizoma Dioscoreae Oppositae*, and *Radix et Rhizoma Clematidis Chinensis.*

Set 2: *Herba Hedyotdis* and *Rhizoma Alismatis.*

Set 3: *Cortex Phellodendri Amurensis*, *Rhizoma Anemarrhenae*, *Radix Angelicae Biserratae*, *Semen Plantaginis*, *Radix Scutellariae Baicalensis*, *Rhizoma Chuanxiong*, and *Fructus Hordei Germinatus.*

### 3.3. Association Rule Analysis of Chinese Herbal Medicine Used in RA Treatment

**Figure 3:**
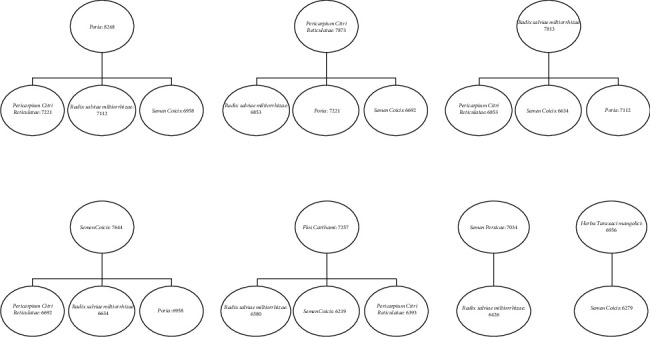
FP-tree of itemset. Note: the number after “:” indicates support of item.

The minimum support and confidence were set to 80%. The Apriori module analysis indicated the correlations among Chinese herbal medicines. The degree of lift was >1 and *P* < 0.05 (Table 2). We set each Chinese herbal medicine as an itemset. We obtained a pair of highly related drugs and the frequency of these highly related drugs (Figure 3).

### 3.4. Improvement of Immune-Inflammatory Indices

Compared with those before treatment, ESR, CRP, IgA, IgG, C3, C4, CCP, and RF decreased significantly in both groups after treatment. After treatment, ESR, CRP, IgA, IgM, IgG, C3, and C4 decreased more significantly in the experimental group than the control group (Table 3).

### 3.5. Evaluation of Immune-Inflammatory Indices by Random Walking Model

**Figure 4:**
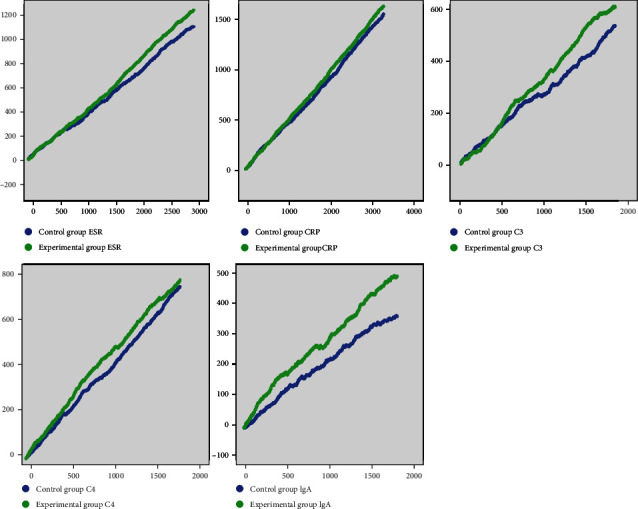
Random walking model of immune-inflammatory indices in RA patients. Note: green line represents the experimental group. Blue line represents the control group. Length of horizontal line increases with the number of walking steps. Height of vertical line increases with intervention efficacy and response.

The ESR of the control and experimental groups had 2,923 and 6,420 comprehensive evaluation records, respectively. The improvement coefficients of the patients were 0.369 and 0.452, respectively. The clinical significance was that the patients had to walk 5.850 and 4.210 steps, respectively, for each comprehensive index improvement. The CRP of both groups had 3,254 and 6,840 comprehensive evaluation records, respectively. The patient improvement coefficients were 0.466 and 0.510, respectively. The clinical significance was that each improvement in the patient comprehensive index required 4.440 and 3.630 steps, respectively. There were 1,795 and 3,430 comprehensive evaluation records for C3 in both groups, respectively. The patient improvement coefficients in both groups were 0.292 and 0.330, respectively. The clinical significance was that for each improvement in the comprehensive index, the patients had to walk 9.880 and 8.040 steps, respectively. There were 1,795 and 3,430 comprehensive evaluation records for C4 in both groups, respectively. The patient improvement coefficients in both groups were 0.416 and 0.432, respectively. The clinical significance was that the patients had to walk 6.930 and 6.140 steps, respectively, for each comprehensive index improvement. There were 1,796 and 3,426 comprehensive evaluation records for IgA in both groups, respectively. The patient improvement coefficients were 0.202 and 0.269, respectively. The clinical significance was that the patients had to walk 14.310 and 9.860 steps, respectively, for each comprehensive index improvement (Table 4 and Figure 4).

## 4. Discussion and Conclusion

Clustering analysis divides similar objects into different sets. Clustering is an unsupervised learning process of searching clusters. The Apriori algorithm clarifies the relationship between items in a dataset. This process is known as a shopping blue analysis. The Apriori algorithm divides association rule discovery into two steps. First, all frequent itemsets in transaction database 1 are retrieved via iteration. The itemsets here are those whose support is not lower than the threshold set by the users. Second, the frequent itemsets are used to construct rules that satisfy minimum user trust. Mining or identifying all frequent itemsets is the core of the algorithm and accounts for most of the computation. A baseline matching algorithm is based on a two-dimensional Euclidean distance. Starting at its minimum value, the target area is tracked and stripped from small to large one unit at a time until it is empty and the objects near the target value are obtained. The random walking model explores the law of motion and integrates the probability and dissipative structure theories [17]. Whether or not there is a long-range correlation in the random walking model, it indicates whether the index system is effective. When long-term correlation is confirmed, the curative effect is measured by calculating the ratio of the random walk cumulative fluctuation value to the random walk point or the random positive increase rate [18]. Here, we used the random walk model to evaluate the therapeutic efficacy of RA drugs.

In traditional Chinese medicine (TCM) theory, RA is in the *Bi syndrome* category. According to TCM theory, *Bi syndrome* occurs in response to incoordination among pathological factors and is mainly attributed to external pathogens such as wind, dampness, heat, and lack of vital body energy. It may manifest spleen deficiency that cannot resist pathological factors [19]. Dampness and wet phlegm transport are weakened in spleen deficiency and gradually progress to humid heat and the stagnated blood stasis syndrome. RA patients may present with joint swelling, chronic pain, fever, joint deformity, and loss of joint function. Hence, we hypothesized that RA pathogenesis comprises spleen deficiency, dampness resistance, heat exuberance, and blood stasis according to TCM theory. The characteristics of TCM include holism and syndrome differentiation-based treatment. However, most patients have a variety of RA symptoms that might change over time. Depending on their symptoms, patients may be classified according to various patterns and treated by different approaches. The use of Chinese herbal medicine in the treatment of the syndrome could alleviate RA symptoms and attenuate side effects caused by chemical drugs [20, 21]. The Chinese herbal medicine administered for RA treatment in our hospital is divided into four categories and can significantly improve RA immunity and inflammation indicators. The efficacy of Chinese herbal medicine combined with prescription drugs is superior to that of Chinese herbal medicine alone [5, 8].

Here, we applied cluster and association rule analyses, baseline matching algorithms, and random walking model data mining to identify Chinese herbal medicine for RA treatment, compatible combinations, and significant therapeutic efficacy against RA.

We identified Chinese herbal medicine commonly used for RA treatment (Table 1). Here, we divided them into four categories according to the efficacy of the constituent herbs. *Poria*, *Pericarpium Citri Reticulatae*, *Semen Coicis*, *Rhizoma Dioscoreae Oppositae*, and *Fructus Hordei Germinatus* were used 33,818 times to invigorate the spleen and resolve dampness. *Radix Salviae Miltiorrhizae*, *Flos Carthami*, *Semen Persicae*, *Caulis Spatholobi*, and *Rhizoma Chuanxiong* were used 28,614 times to promote blood circulation and dredge collaterals. *Radix et Rhizoma Clematidis Chinensis*, *Sigesbeckia orientalis L.*, *Rhizoma Alismatis*, *Semen Plantaginis*, and *Radix Angelicae Biserratae* were used 20,063 times to dispel wind and dehumidify. *Herba Taraxaci Mongolici*, *Herba Hedyotdis*, *Radix Scutellariae Baicalensis*, *Cortex Phellodendri Amurensis*, and *Rhizoma Anemarrhenae* were applied for heat clearing and detoxification. Within the four classes of Chinese herbal medicine, the spleen meridian was used 35,478 times, sweet taste was used 57,052 times, and bitter taste was used 50,762 times. In traditional Chinese medicine, bitter taste is used to dehumidify while sweet taste is used to tonify the spleen. Both can verify that RA pathogenesis originates mainly from spleen deficiency and dampness. Data mining technology disclosed that the Chinese herbal medicines most efficacious at treating RA were those that invigorated the spleen, resolved dampness, cleared heat, and dredged collaterals. Thus, administration of these preparations may reverse RA pathogenesis. The main pathogenic factors are wind, dampness, heat, spleen deficiency, and collateral stasis caused by RA joint symptoms.

By cluster analysis, we extracted common Chinese herbal medicine combinations for RA treatment (Figure 2). We conducted a cluster analysis on commonly used Chinese herbal medicines to discover various combination rules for their use. Chinese herbal medicines were divided into three sets. Herbs in the first set invigorate the spleen, remove dampness, and dredge collaterals. The herbs in the second set clear heat and dampness. The herbs in the third set clear heat, promote dampness, invigorate the spleen, and dredge collaterals.

To elucidate the compatibility of the herbs commonly used for RA treatment in our hospital, we analyzed their association rules (Tables 2; Figure 3). This process determines the degree of support and confidence. We set the minimum support and confidence to 80%, the degree of improvement to >1, and *P* < 0.05. We obtained one pair of highly correlated drugs and the frequency of these highly correlated drugs. Herb compatibility clarification is invaluable in planning rational clinical drug use, enhancing curative efficacy, and developing modern pharmacy.

The efficacy of Chinese herbal medicine at treating RA has been confirmed (Tables 3 and 4; Figure 4). In Asian countries, compatible Chinese medicines have been widely used in clinical RA treatment as the combinations are simple, flexible, and efficient [22]. In the present study, we applied data mining technology to identify the rules of use of Chinese herbal medicine for RA treatment in our hospital and tested the efficacy of herbal medicine in RA treatment. Xinfeng capsule (Anhui medicine No. Z20050062) is a prescription hospital preparation of Anhui Traditional Chinese Medicine Hospital. It consists of *Radix Astragali Mongolici*, *Semen Coicis*, *Radix et Rhizoma Tripterygii*, and *Scolopendra*. It invigorates the spleen, replenishes qi, resolves dampness, removes arthralgia, promotes blood circulation, and removes meridian obstructions. It is a traditional Chinese medicine prescription with a long history of use in clinical RA treatment. Furong ointment is a prescription preparation of our hospital. It clears heat, detoxifies, reduces swelling, relieves pain, and promotes healing in RA.

Of the 9,408 RA patients in this study, 3,533 were in the control group and 5,875 were in the experimental group. The degree of immune-inflammatory response varied among RA patients and was reflected mainly in the differences in the numerical values of their immune-inflammatory indices. We matched the immune-inflammatory indices of both groups by a computer matching algorithm before treatment. In this manner, we unified the disease before therapy. After treatment, the immune-inflammatory indices of both groups significantly decreased. Compared with the control group, ESR, CRP, IgA, IgG, C3, and C4 were significantly lower in the experimental group. Hence, there was strong therapeutic efficacy of Chinese herbal decoctions combined with prescription preparations.

We also applied a random walking model to evaluate the immune-inflammatory indices of both groups of RA patients. The improvement coefficients of ESR, CRP, C3, C4, and IgA were higher for the experimental group than those for the control group. There were fewer walking steps in the experimental group than the control group for the improvement of each comprehensive index. Thus, there is a long-term correlation between RA treatment and Chinese herbal decoctions combined with prescription preparations. Further, the combination had a superior curative effect to that of the Chinese herbal decoction alone.

RA pathogenesis is complex. Compatible Chinese herbal medicines administered for RA treatment have numerous pharmacological effect targets [23]. The commonly used Chinese herbal medicine we extracted had a positive therapeutic effect on RA. The 2015 edition of the Chinese Pharmacopoeia lists the functions of commonly used Chinese herbal medicines including spleen invigoration, dampness resolution, blood circulation promotion, blood stasis removal, heat clearing, and detoxification. These findings are consistent with RA pathogenesis in traditional Chinese medicine, and these Chinese herbal medicines have been widely used in RA treatment [24].

The data mining method used here has several advantages. First, it requires no data structures. This property is very useful for data mining in Chinese herbal medicine as the data structure for most Chinese herbal medicines is not uniform. Second, a variety of data mining methods are applied for comprehensive analyses. In this way, reliability of the results is assured. Third, we can verify the clinical efficacy of the extracted Chinese herbal medicine to ensure the accuracy of the conclusion. The most important aspect of data mining technology is that it facilitates learning the main treatment methods and the uses of Chinese herbal medicine. Our study also has some limitations. We collected only prescription information but not diagnostic information of TCM syndrome classification, so our results have certain deviations. Besides, the safety of the drugs was not evaluated and should be investigated in future research.

In conclusion, we applied clustering analysis, association rules, a baseline matching algorithm, and a random walking model to show that there are complementary relationships among the constituent herbs in Chinese herbal medicine used to treat RA. Various combinations of these materials produce different therapeutic effects in RA treatment. The new clinical evaluation method known as random walking dynamically evaluates the therapeutic efficacy of drugs against RA and provides a novel technique for evaluating clinically applied medications.

## Figures and Tables

**Table 1:** Use of Chinese herbal medicine in RA treatment.

Category	Herb	Number	Property and taste	Meridian tropism
Invigorating spleen and resolving dampness	*Poria*	8,248	Sweet, light, flat	Spleen, kidney
	*Pericarpium Citri Reticulatae*	7,873	Pungent, bitter, warm	Spleen, lung
	*Semen Coicis*	7,644	Sweet, light, cool	Spleen, stomach
	*Rhizoma Dioscoreae Oppositae*	6,926	Sweet, flat	Spleen, lung, kidney
	*Fructus Hordei Germinatus*	2,527	Sweet, flat	Spleen, stomach, liver

Promoting blood circulation to dredge collaterals	*Radix Salviae Miltiorrhizae*	7,813	Bitter, slightly cold	Heart, liver
	*Flos Carthami*	7,257	Pungent, warm	Heart, liver
	*Semen Persicae*	7,034	Bitter, sweet, flat	Heart, liver
	*Caulis Spatholobi*	3,992	Bitter, slightly sweet, warm	Liver, kidney
	*Rhizoma Chuanxiong*	2,518	Pungent, warm	Liver

Wind-dispelling and dehumidification	*Radix et Rhizoma Clematidis Chinensis*	6,189	Pungent, salt, warm	Bladder
	*Sigesbeckia orientalis*	5,247	Pungent, bitter, cold	Kidney, liver
	*Rhizoma Alismatis*	4,869	Sweet, cold	Kidney, bladder
	*Semen Plantaginis*	2,383	Sweet, slightly cold	Liver, kidney, lung, small intestine
	*Radix Angelicae Biserratae*	1,375	Pungent, bitter, warm	Kidney, bladder

Heat clearing and detoxification	*Herba Taraxaci Mongolici*	6,956	Bitter, sweet, cold	Liver, stomach
	*Herba Hedyotdis*	4,890	Bitter, sweet, cold	Stomach, larger intestine
	*Radix Scutellariae Baicalensis*	2,260	Bitter, cold	Lung, stomach, larger intestine
	*Cortex Phellodendri Amurensis*	1,739	Bitter, cold	Kidney, bladder
	*Rhizoma Anemarrhenae*	1,583	Bitter, sweet, cold	Lung, stomach

Values indicate the number of times the drug is used. Invigorating spleen and resolving dampness: herbs have efficacy to invigorate the spleen and resolve dampness. Promoting blood circulation to dredge collaterals: herbs have efficacy to promote blood circulation and dredge collaterals. Wind-dispelling and dehumidification: herbs have efficacy to dispel wind and dehumidify. Heat clearing and detoxification: herbs have efficacy to clear heat and detoxify.

**Table 2:** Correlation of Chinese herbal medicine in RA treatment.

Items (LHS⇒RHS)	Support	Confidence	Expected confidence	Lift	*P* value
{*Pericarpium Citri Reticulatae*}⇒{*Poria*}	83.68%	91.72%	87.67%	1.05	<0.01
{*Radix Salviae Miltiorrhizae*}⇒{*Poria*}	83.05%	91.03%	87.67%	1.04	<0.01
{*Semen Coicis*}⇒{*Poria*}	81.25%	91.03%	87.67%	1.04	<0.01
{*Radix Salviae Miltiorrhizae*}⇒{*Pericarpium Citri Reticulatae*}	83.05%	87.71%	83.68%	1.05	<0.01
{*Poria*}⇒{*Pericarpium Citri Reticulatae*}	87.67%	87.55%	83.68%	1.05	<0.01
{*Semen Coicis*}⇒{*Pericarpium Citri Reticulatae*}	81.25%	87.55%	83.68%	1.05	<0.01
{*Pericarpium Citri Reticulatae*}⇒{*Radix Salviae Miltiorrhizae*}	83.68%	87.04%	83.05%	1.05	<0.01
{*Semen Coicis*}⇒{*Radix Salviae Miltiorrhizae*}	81.25%	86.79%	83.05%	1.05	<0.01
{*Poria*}⇒{*Radix Salviae Miltiorrhizae*}	87.67%	86.23%	83.05%	1.04	<0.01
{*Pericarpium Citri Reticulatae*}⇒{*Semen Coicis*}	83.68%	85.00%	81.25%	1.05	<0.01
{*Radix Salviae Miltiorrhizae*}⇒{*Semen Coicis*}	83.05%	84.91%	81.25%	1.05	<0.01
{*Poria*}⇒{*Semen Coicis*}	87.67%	84.36%	81.25%	1.04	<0.01
{*Radix Salviae Miltiorrhizae*}⇒{*Flos Carthami*}	83.05%	84.22%	77.14%	1.09	<0.01
{*Radix Salviae Miltiorrhizae*}⇒{*Semen Persicae*}	83.05%	82.25%	74.77%	1.10	<0.01
{*Semen Persicae*}⇒{*Herba Taraxaci Mongolici*}	81.25%	82.14%	73.94%	1.11	<0.01
{*Semen Coicis*}⇒{*Flos Carthami*}	81.25%	81.36%	77.14%	1.06	<0.01
{*Pericarpium Citri Reticulatae*}⇒{*Flos Carthami*}	83.68%	81.20%	77.14%	1.05	<0.01

Values are % degrees of relevancy.

**Table 3:** Improvement of immune-inflammatory indices.

Index	Control group		Experimental group		*P*_2_ value
	*d*_0_	*P*_0_ value	*d*_1_	*P*_1_ value	
ESR (mm/h)	-24.213	0.00	-27.964	0.00	0.000
CRP (mg/L)	-31.166	0.00	-33.468	0.00	0.000
IgA (g/L)	-11.682	0.00	-13.162	0.00	0.027
IgM (g/L)	-1.004	0.32	-4.466	0.00	0.017
IgG (g/L)	-13.731	0.00	-16.249	0.00	0.003
C3 (g/L)	-15.286	0.00	-17.495	0.00	0.035
C4 (g/L)	-20.878	0.00	-23.099	0.00	0.000
CCP (mmol/L)	-4.352	0.00	-3.352	0.00	0.333
RF (U/mL)	-16.528	0.00	-15.815	0.00	0.432

Note: ESR: erythrocyte sedimentation rate; CRP: C-reactive protein; IgA: immunoglobulin A; IgM: immunoglobulin M; IgG: immunoglobulin G; C3: complement C3; C4: complement C4; CCP: anticyclic citrullinated peptide; RF: rheumatoid factor. *d*_0_ is the difference in the control group before and after treatment. *P*_0_ is the comparison between the control group before and after treatment. *d*_1_ is the difference in the experimental group before and after treatment. *P*_1_ is the comparison between the experimental group before and after treatment. *P*_2_ is comparison between both groups after treatment.

**Table 4:** Random walking model of immune-inflammatory indices.

Index	Group	Maximum random fluctuation	Walking positive growth rate	Random fluctuation power law value	Improvement coefficient	Comprehensive evaluation records	Ratio
ESR	Control group	1,078	0.171	0.398 ± 0.106	0.369	2,923	5.850
	Experimental group	2,899	0.238	0.483 ± 0.103	0.452	6,420	4.210
CRP	Control group	1,515	0.225	0.427 ± 0.110	0.466	3,254	4.440
	Experimental group	3,490	0.275	0.412 ± 0.084	0.510	6,840	3.630
C3	Control group	524	0.101	0.370 ± 0.106	0.292	1,795	9.880
	Experimental group	1,131	0.124	0.417 ± 0.110	0.330	3,430	8.040
C4	Control group	747	0.144	0.331 ± 0.113	0.416	1,795	6.930
	Experimental group	1,482	0.163	0.399 ± 0.111	0.432	3,430	6.140
IgA	Control group	362	0.007	0.359 ± 0.078	0.202	1,796	14.310
	Experimental group	922	0.101	0.397 ± 0.069	0.269	3,426	9.860

Note: ESR: erythrocyte sedimentation rate; CRP: C-reactive protein; C3: complement C3; C4: complement C4; IgA: immunoglobulin A.

## Data Availability

The datasets generated for this study are available on request to the corresponding authors.
